# The role of Na_v_1.9 channel in the development of neuropathic orofacial pain associated with trigeminal neuralgia

**DOI:** 10.1186/s12990-015-0076-4

**Published:** 2015-11-25

**Authors:** Ana Paula Luiz, Olga Kopach, Sonia Santana-Varela, John N. Wood

**Affiliations:** Molecular Nociception Group, Wolfson Institute for Biomedical Research, University College London, Gower St, London, WC1E 6BT UK; Department of Molecular Medicine and Biopharmaceutical Sciences, College of Medicine, Seoul National University, Seoul, South Korea

**Keywords:** Na_v_1.9 sodium channels, Trigeminal ganglion (TG) neurons, Constriction of the infraorbital nerve, Trigeminal neuralgia, Neuropathic pain

## Abstract

**Background:**

Trigeminal neuralgia is accompanied by severe mechanical, thermal and chemical hypersensitivity of the orofacial area innervated by neurons of trigeminal ganglion (TG). We examined the role of the voltage-gated sodium channel subtype Na_v_1.9 in the development of trigeminal neuralgia.

**Results:**

We found that Na_v_1.9 is required for the development of both thermal and mechanical hypersensitivity induced by constriction of the infraorbital nerve (CION). The CION model does not induce change on Na_v_1.9 mRNA expression in the ipsilateral TG neurons when evaluated 9 days after surgery.

**Conclusions:**

These results demonstrate that Na_v_1.9 channels play a critical role in the development of orofacial neuropathic pain. New routes for the treatment of orofacial neuropathic pain focussing on regulation of the voltage-gated Na_v_1.9 sodium channel activity should be investigated.

## Background

Trigeminal neuralgia is an excruciating pain syndrome characterized by severe facial pain that can be triggered by light touch of the orofacial surface area producing stabbing, shooting or burning sensations. Trigeminal neuralgia is accompanied by mechanical, thermal and/or chemical hypersensitivities of the orofacial area and is maintained by impaired signaling in sensory neurons of trigeminal ganglion (TG) [1]. However, little is known about the intracellular mechanisms or altered functioning of TG nociceptors underling the development of orofacial pain.

Voltage-gated sodium channels play a central role in nociception, being key determinants of neuronal excitability. Specific expression of tetrodotoxin-resistant (TTX-R) voltage-gated sodium channels Na_v_1.8 and Na_v_1.9 subtypes was found for a restricted population of peripheral nociceptors in TG [2–4], dorsal root ganglion (DRG) [5–9], nodose ganglion [10], unmyelinated afferent fibres and nerve terminals [8, 9, 11, 12]. While Na_v_1.8 channels carry the majority of inward current during the upstroke of the action potentials (AP) [13–15], Na_v_1.9 channels sustain repetitive firing and plateau potentials [16, 17] due to their characteristic activation at relatively negative membrane potentials as well as slow and incomplete inactivation, producing “persistent” Na^+^ flow at subthreshold voltages [18–20].

Studies of the role of Na_v_1.9 channels showed that SCN11A null mutant mice developed reduced hyperalgesia in different inflammatory pain models [formalin, carrageenan, complete freund’s adjuvant (CFA), prostaglandin E_2_] [21, 22] as well as diminished nociceptive sensitivity triggered by inflammatory mediators [bradykinin, serotonin, adenosine triphosphate (ATP)] [2]. In contrast, Na_v_1.9^−/−^ mice showed unaltered pain-related behaviour in models of DRG-related neuropathic pain of various origins (partial sciatic nerve injury [2], chronic constriction injury (CCI) [23] and spinal nerve transaction [24]). Recent studies of human SCN11A gene mutations demonstrated that several mutations in this gene are associated with either, peripheral neuropathy and episodic chronic pain [25] or congenital inability to experience pain [26]. Despite the established role of Na_v_1.9 channels in pain perception, the contribution of this channel subtype to the development of orofacial pain remains unknown, and how cephalic neuropathic pain develops with loss of Na_v_1.9 channels has not been studied.

Using global Na_v_1.9 knockout mice, we show for the first time that loss of Na_v_1.9 channels is associated with the failure to develop orofacial pain in a model of trigeminal neuralgia.

## Results

### Basal orofacial sensitivity in Na_v_1.9^+/+^ and Na_v_1.9^−/−^ mice

**Fig. 1:**
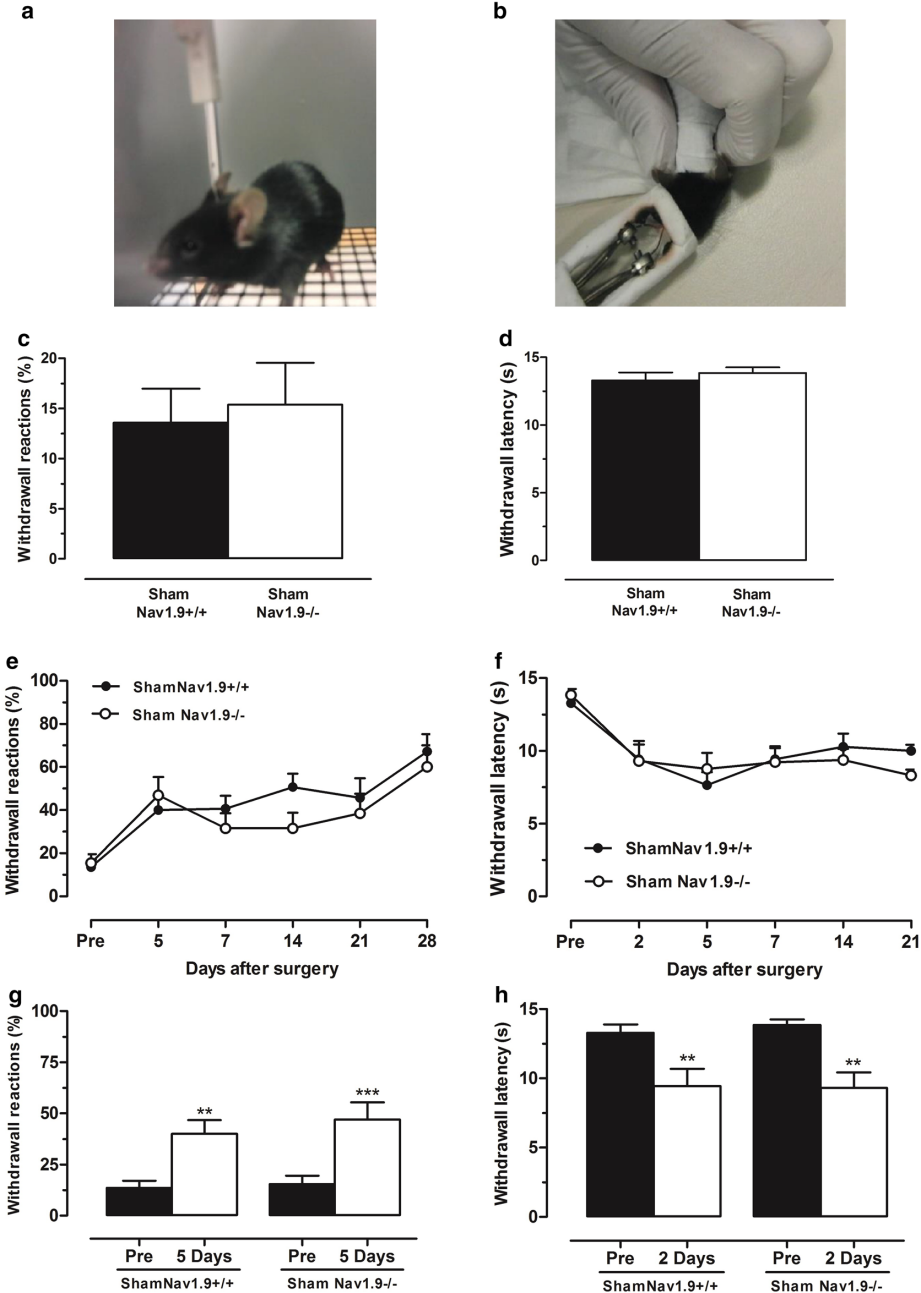
Loss of Na_v_1.9 does not influence orofacial sensitivity in mice. Mechanical orofacial sensitivity in response to 0.04 g von Frey filament applied to the forehead (**a**) and thermal orofacial sensitivity in response to radiant heat applied to a vibrissal pad (**b**) in sham-operated Na_v_1.9^+/+^ and Na_v_1.9^−/−^ mice. Mechanical (**c**) and thermal (**d**) orofacial sensitivities baselines. Mechanical (**e**) and thermal (**f**) orofacial sensitivities evaluated before (pre) and at different time-points after surgery. Changes in mechanical (**g**) and thermal (**h**) orofacial sensitivities after sham-operation. Values represent mean ± SEM analysed for 13–14 mice. **p < 0.01, ***p < 0.001 compared to that before surgery (pre) (Student paired t test)

To examine if the loss of Na_v_1.9 channels could influence the sensitivity of the orofacial area, basal mechanical and thermal sensitivities were examined in Na_v_1.9^+/+^ and Na_v_1.9^–/–^ mice. Mechanical orofacial sensitivity was evaluated as a functional reaction in response to 0.04 g von Frey filament applied to the animal forehead innervated by the trigeminal nerve (Fig. 1a). A filament of 0.04 g intensity was chosen since I have been found to evoke nociceptive responses in the orofacial area after unilateral constriction of the infraorbital nerve (CION), but present almost no effect in control mice [27]. No differences in basal responses to mechanical stimulation were observed between genotypes (n = 13–14 mice, p > 0.05; Fig. 1c). Groups did not differ with regard to basal thermal sensitivity, measured as a latency of response to radiant heat applied to the vibrissal pad surface (n = 13–14 mice, p > 0.05; Fig. 1b, d). Thus, loss of Na_v_1.9 channels does not affect basal orofacial sensitivity, either mechanical or thermal, in mice.

We next examined the responses of control and knockout mice to a sham surgical inflammation without nerve damage. No major differences in mechanical and thermal sensitivities were found between Na_v_1.9^+/+^ and Na_v_1.9^–/–^ mice, submitted to this sham surgical procedure with no CION, at different time-points (on days 2, 5, 7, 14, 21 and 28 post-surgery; n = 13–14 mice; Fig. 1e, f). Although, the orofacial area was sensitised after the sham surgical procedure, the surgery-induced changes were similar between Na_v_1.9^+/+^ and Na_v_1.9^−/−^ sham-operated mice for either threshold mechanical (by 26 %, p < 0.01 and by 31 %, p < 0.001 in Na_v_1.9^+/+^ and Na_v_1.9^−/−^ mice, respectively; n = 13–14 mice; Fig. 1g) or threshold thermal sensitivities (by 29 % and by 31 %, n = 13–14 mice, p < 0.01 in Na_v_1.9^+/+^ and Na_v_1.9^−/−^ mice, respectively; Fig. 1h).

### Loss of Na_v_1.9 channels alleviates CION-induced orofacial hypersensitivity

**Fig. 2:**
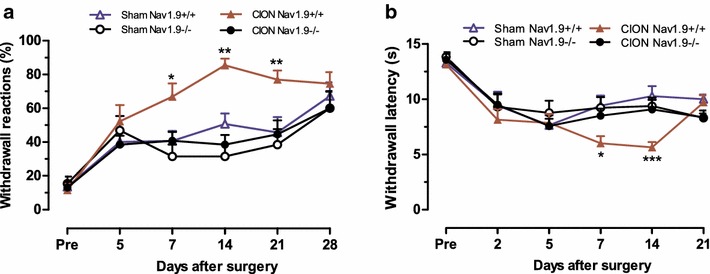
Orofacial neuropathic hypersensitivity was attenuated in Na_v_1.9 KO mice. Changes in mechanical orofacial sensitivity in response to 0.04 g von Frey filament applied to the forehead (**a**) and thermal orofacial sensitivity in response to radiant heat applied to a ipsilateral vibrissal pad (**b**) in Na_v_1.9^+/+^ and Na_v_1.9^−/−^ mice before surgery (pre) and at different time-point after CION. Values represent mean ± SEM. analysed for 13–14 mice. *p < 0.05, **p < 0.01, ***p < 0.001 compared to the correspondent time-point in sham Na_v_1.9^+/+^ mice (Two-way ANOVA followed by Bonferroni’s test)

Constriction of the infraorbital nerve [25] led to a development of long-lasting mechanical hypersensitivity in Na_v_1.9^+/+^. This hypersensitivity developed 7 days after surgery, reached a peak at 2 weeks, and remained persistent until 3 weeks (Fig. 2a). CION-induced mechanical hypersensitivity was robust, showing a significantly increased reaction in response to von Frey filament applied to the forehead of Na_v_1.9^+/+^ and Na_v_1.9^−/−^ mice. The increase in the response frequency was 26 % (p < 0.05), 35 % (p < 0.01) and 31 % (p < 0.01) on day 7, 14 and 21 after CION respectively compared to sham-operated mice (n = 13–14 mice; Fig. 2a). Strikingly, in Na_v_1.9^−/−^ mice, CION was not accompanied by the development of hypersensitivity of the orofacial area. The responses of Na_v_1.9^−/−^ mice were similar to those observed in the littermates of the sham-operated group over all periods tested. Thus, loss of Na_v_1.9 channels abolished the development of CION-induced mechanical hypersensitivity.

Loss of Na_v_1.9 channels also abolished the development of CION-induced thermal hypersensitivity. Consistent with previous studies [28], CION produced a long-lasting thermal hypersensitivity in Na_v_1.9^+/+^ mice, which developed within 1 week after surgery. The CION-induced decrease in thermal latency in Na_v_1.9^+/+^ mice was 10 % (p < 0.05) on day 7 and 12 % (p < 0.001) on day 14 post-surgery. However, the latency was not changed in Na_v_1.9^−/−^ mice following CION over all period tested (n = 13–14 mice; Fig. 2b). Together, these results indicate that the Na_v_1.9 channel is required for the development of neuropathic orofacial pain.

### Expression of Na_v_1.9 mRNA in TG on Sham and CION Na_v_1.9^+/+^ mice

**Fig. 3:**
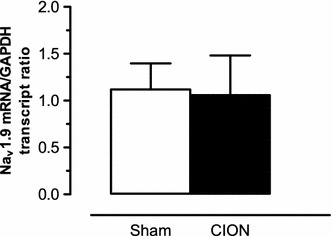
Expression of Na_v_1.9 mRNA in TG on Sham and CION Na_v_1.9^+/+^ mice 9 days after surgery. Values represent mean ± SEM analysed for 6–7 mice (Student paired t test)

As the Na_v_1.9^−/−^ mice did not develop mechanical or thermal hypersensitivity after CION, the next step was to evaluate if the CION model induced any change in the expression of Na_v_1.9 mRNA in TG neurons. Sample from ipsilateral TG was processed on day 9 after surgery, for extraction of mRNA. As showed on Fig. 3, no Na_v_1.9 mRNA expression difference was observed between the CION (1.06 ± 0.42) and Sham (1.12 ± 0.28) groups in Na_v_1.9^+/+^ mice (p = 0.9026).

## Discussion

Considerable evidence suggests participation of Na_v_1.9 channels in the perception of pain. Both gain of function and loss of function pain conditions have been associated with point mutations in Na_v_1.9 [25, 26]. Two of those mutations led to a reduction in the current threshold and increased firing frequency in response to suprathreshold stimuli [25]. Mutations in SCN11A (R225C and A808G), reported in patients experienced episodic chronic pain, caused an increase in the Na_v_1.9 channels-mediated current density and hyperexcitability of nociceptive DRG neurons without changes in the resting membrane potential [29]. Other gain of function mutations lead Na_v_1.9 channels to display excessive activity at resting voltages, causing sustained depolarization of nociceptors, impaired generation of action potentials and resulting in a pain-free state [26]. The principal mechanism by which Na_v_1.9 channels contribute to painful states includes sensitization of nociceptors triggered by generation of persistent Na^+^ currents at subthreshold voltages that promotes depolarization near the resting membrane potential, reduces the current threshold required to trigger an action potential, and results in hyperexcitability of sensory neurons [7, 16, 18–20, 30]. Here using Na_v_1.9^−/−^ mice, we show that Na_v_1.9 channels are also involved in sustained (tonic) firing of nociceptive TG neurons and are required for the development of orofacial neuropathic pain.

The role of the Na_v_1.9 channel in maintaining peripheral inflammatory pain has been demonstrated in different studies [21, 22, 30]. Inflammatory pain of different origins (induced by formalin, carrageenan, CFA, capsaicin, ATP, IL-1β or prostaglandin-E2) was substantially reduced in Na_v_1.9^−/−^ mice [21, 22, 30], while neuropathic pain, produced by CCI or spinal nerve transection, still developed, showing mechanical allodynia of the hind paw of Na_v_1.9^−/−^ mice [24, 31]. Using CION-induced model of orofacial neuropathic pain [28, 32], we demonstrate here the absence of both mechanical and thermal hypersensitivity of the orofacial area in Na_v_1.9^−/−^ mice after CION. Loss of Na_v_1.9 channels did not change basal orofacial sensitivity (mechanical or thermal), indicating no major effects of Na_v_1.9 deletion on normal nociceptive pathways. Our findings of a crucial role for Na_v_1.9 channels in the development of neuropathic orofacial pain are in contrast with reports showing no critical involvement of these channels in the development of neuropathic pain in other somatic neuropathic pain models [24, 31]. This may reflect distinct mechanisms of neuropathic pain.

Na_v_1.9 channels enhance sustained repetitive (tonic) firing in TG neurons. Previous studies showing that Na_v_1.9 channels maintain sustained repetitive firing in DRG nociceptors and modulate patterns of firing discharge, including plateau potentials, active hyperpolarizing responses, and sustained oscillatory bursting discharges [16, 18, 33]. It is intriguing that gain of function Na_v_1.9 mutations in humans may result in enhanced pain or a complete loss of pain sensation [25, 26]. In transgenic mice, inflammatory pain is associated with enhanced Na_v_1.9 persistent sodium channel activity [30]. In the present study, neuropathic pain is dependent on the activity of Na_v_1.9 in a model of trigeminal neuralgia. A critical role for Na_v_1.9 in regulating pain thresholds is thus clear, but the precise mechanisms that contribute to the different pain phenotypes remains to be established.

## Conclusions

Our study demonstrates for the first time that the Na_v_1.9 channel plays a critical role in the development of neuropathic orofacial pain associated with trigeminal neuralgia. Understanding how orbital nerve damage leads to myofascial pain through the modulation of Na_v_1.9 activity may provide new approaches to the treatment of trigeminal neuralgia.

## Methods

### Animals

C57Bl/6 mice (2–3 month-old) were used in accordance with the protocols approved by the UK Home Office and UCL ethics committee under a Home Office project license. All efforts were made to minimise animal suffering and to reduce the number of animals used. Experiments were conducted using both male and female wild type littermate and global Na_v_1.9 knockout mice bred from heterozygous (Na_v_1.9^+/−^) animals. The generation and characterization of the Na_v_1.9 null mutant line was previously described [34]. Mouse colonies were genotyped by PCR using ear biopsy samples. Only homozygous Na_v_1.9-null (Na_v_1.9^−/−^) and wild type (Na_v_1.9^+/+^) mice were used in these studies. Genomic DNA was extracted using a lysis buffer containing (in mM) 67 Tris, 16 (NH_4_)_2_SO_4_, 6.7 MgCl_2_, 100 β-mercaptoethanol, and 0.5 % Triton X-100 and 0.05 mg/ml proteinase K. The primers used were: 5′-AACAGTCTTACGCTGTTCCGATG-3′ (sense), 5′-ATGTGGCACTGGGCTTGAACTC-3′ (antisense), 5′-CTCGTCGTGACCCATGGCGAT-3′ (Neomycin FW). PCR was performed in one reaction and resulted in a 276 bp fragment product for WT and a 600 bp band for knockout mice.

### Infraorbital nerve constriction

To produce the painful condition of trigeminal neuralgia, we used a model of unilateral CION [25] according to a method previously described for rats [28, 35] with some modifications. Briefly, mice were anesthetized with an intramuscular (i.m.) injection of a mixture of ketamine (Fort Dodge Animal Health LTD, UK) and medetomidine (Orion Pharma, UK) in the doses of 50 and 0.5 mg/kg, respectively. One incision was made below the right eye 1 mm caudal to the myofascial (vibrissal) pads. The superior lip elevator and anterior superficial masseter muscles were dissected to expose the rostral end of the infraorbital nerve, as it emerges from the infraorbital fissure. Extra care was taken to prevent facial nerve damage. Two silk ligatures (No. 8.0, Ethicon) were loosely tied at a distance of 2 mm around the infraorbital nerve, producing a development of orofacial hypersensitivity and preventing infraorbital nerve destruction [35]. The incision was closed with polypropylene sutures (No. 6.0, Ethicon). After surgery, all animals were injected i.m. with atipamezole (5 mg/kg, Orion Pharma) and were maintained in a warmed area until full recovery from anaesthesia. Animals operated on an identical manner with no ligature applied to the nerve were used as control (sham).

### Behavioral tests

#### Measurement of mechanical hyperalgesia

Mice were acclimatized in an individual animal enclosure (4″ × 4″) chamber for at least 1 h. Sham-operated or CION-injured animals were submitted to a repeated mechanical stimulus (0.04 g von Frey filament) applied to the forehead surface innervated by the trigeminal nerve (Fig. 1a). Each trial was repeated 10 times at 30-s interval at least. The occurrence of head reactions (attack/escape or head withdrawal) was expressed for each trial as the mean of percentage reactions that represents an index of mechanical nociceptive sensitivity [27]. To determine basal mechanical sensitivity, the mechanical stimulus was applied to the forehead of animals 1 day before surgery. A development of mechanical hyperalgesia following CION was estimated at different days after surgery.

#### Measurement of thermal hyperalgesia

Thermal hyperalgesia of the orofacial area was measured as previously described [28, 36]. Each animal was held in front of a radiant heat source positioned 1 cm from the vibrissal pad surface (Fig. 1b). The intensity of thermal stimulus applied to the vibrissal pad skin was adjusted to temperature of ~50 °C (15 s cut off). Once the animal started to withdraw its head or vigorously flick a snout, the heat beam was turned off. The time between the start of the beam and a functional response was defined as a latency of response. A reduction in the response latency reflected thermal hyperalgesia. To determine basal thermal sensitivity, a heat stimulus was applied to the ipsilateral side of the snout 1 day before surgery. Basal latency of responses was typically from 10 to 15 s. The development of thermal hyperalgesia following CION was measured at different days after surgery.

### Na_v_1.9 expression analysis real-time PCR

After animal euthanasia (with a CO_2_ chamber following by cervical dislocation), TG was quickly removed and placed in buffer RTL and RNA was isolated using RNeasy mini kit (Quiagen) according to the manufacturer instructions. The RNA was quantified by Nanodrop and converted to cDNA using iScript™ reverse transcription supermix for qRT-PCR (BioRad) according to the manufacturer instructions. PCR amplifications were performed using a Mastercycler ep realplex (Eppendorf). All samples were run in triplicate in a final volume of 20 µl containing 10 ng of cDNA, 1 µM of primers and SsoAdvanced™ universal SYBR^®^ Green supermix (BioRad), according to manufacturer protocol. Prior to PCR, an 8 min enzyme activation step was done at 95 °C. The PCR protocol consisted of 10 s denaturation at 95 °C, 5 s at annealing temperature at 63 °C, 10 s elongation at 72 °C for 40 cycles. The annealing temperature was confirmed by melting curve. The primers sequences used were the following: TTCCACTCTACGTACCTTCCGAGT (forward) and ATTCCCATGAAGAGCTGCTGACCA (reverse) for Na_v_1.9; TGCGACTTCAACAGCAACTC (forward) and CTTGCTCAGTGTCCTTGCTG (reverse) for GAPDH.

Na_v_1.9 and GAPDH cDNA relative amounts were calculated function of the samples cycle threshold (Ct) using a standard concentration curve constructed with a serial dilution (10 times) from 100 to 0.01 ng of cDNA mix. For each sample, the relative amount of Na_v_1.9 cDNA was normalized by the GAPDH cDNA amount.

### Statistical analysis

Data were analyzed using GraphPad Prism 5. The results are presented as mean ± SEM with *n* referring to the number of animals tested. Multiple groups were compared using two-way analysis of variance [37] followed by Bonferroni post hoc test or Student paired t test as indicated.

Values of p less than 0.05 was considered as statistically significant for either test used.

## Abbreviations

AP: action potential
ATP: adenosine triphosphate
CCI: chronic constriction injury
CFA: complete freund adjuvant
CION: constriction of the infraorbital nerve
DRG: dorsal root ganglion
IL-1β: interleukin-1 beta
mRNA: messenger ribonucleic acid
Nav1.8: voltage-gated sodium channel 1.8 subtype
Nav1.9: voltage-gated sodium channel 1.9 subtype
qRT-PCR: quantitative reverse transcription polymerase chain reaction
SCN11A: gene coding voltage-gated sodium channel 1.9 subtype
TG: trigeminal ganglion
TTX-R: tetrodotoxin-resistant
